# Automatic Segmentation of Mediastinal Lymph Nodes and Blood Vessels in Endobronchial Ultrasound (EBUS) Images Using Deep Learning

**DOI:** 10.3390/jimaging10080190

**Published:** 2024-08-06

**Authors:** Øyvind Ervik, Ingrid Tveten, Erlend Fagertun Hofstad, Thomas Langø, Håkon Olav Leira, Tore Amundsen, Hanne Sorger

**Affiliations:** 1 Clinic of Medicine, Nord-Trøndelag Hospital Trust, Levanger Hospital, 7601 Levanger, Norway; hanne.sorger@ntnu.no; 2 Department of Circulation and Medical Imaging, Faculty of Medicine and Health Sciences, Norwegian University of Science and Technology, 7030 Trondheim, Norway; hakon.o.leira@ntnu.no (H.O.L.); tore.amundsen@ntnu.no (T.A.); 3 Department of Health Research, SINTEF Digital, 7034 Trondheim, Norway; ingrid.tveten@sintef.no (I.T.); erlend.hofstad@sintef.no (E.F.H.); thomas.lango@sintef.no (T.L.); 4 Department of Research, St. Olavs Hospital, 7030 Trondheim, Norway; 5 Department of Thoracic Medicine, St Olavs Hospital, Trondheim University Hospital, 7030 Trondheim, Norway

**Keywords:** bronchoscopy, endobronchial ultrasound, deep learning, deep neural networks, segmentation

## Abstract

Endobronchial ultrasound (EBUS) is used in the minimally invasive sampling of thoracic lymph nodes. In lung cancer staging, the accurate assessment of mediastinal structures is essential but challenged by variations in anatomy, image quality, and operator-dependent image interpretation. This study aimed to automatically detect and segment mediastinal lymph nodes and blood vessels employing a novel U-Net architecture-based approach in EBUS images. A total of 1161 EBUS images from 40 patients were annotated. For training and validation, 882 images from 30 patients and 145 images from 5 patients were utilized. A separate set of 134 images was reserved for testing. For lymph node and blood vessel segmentation, the mean ± standard deviation (SD) values of the Dice similarity coefficient were 0.71 ± 0.35 and 0.76 ± 0.38, those of the precision were 0.69 ± 0.36 and 0.82 ± 0.22, those of the sensitivity were 0.71 ± 0.38 and 0.80 ± 0.25, those of the specificity were 0.98 ± 0.02 and 0.99 ± 0.01, and those of the F1 score were 0.85 ± 0.16 and 0.81 ± 0.21, respectively. The average processing and segmentation run-time per image was 55 ± 1 ms (mean ± SD). The new U-Net architecture-based approach (EBUS-AI) could automatically detect and segment mediastinal lymph nodes and blood vessels in EBUS images. The method performed well and was feasible and fast, enabling real-time automatic labeling.

## 1. Introduction

Lung cancer is the leading cause of cancer-related deaths worldwide [1]. A patient’s potential for a cancer cure and long-term survival largely depend on the stage of the disease [2,3]. Endobronchial ultrasound (EBUS) utilizes ultrasound technology to visualize structures within the airways and surrounding areas, serving as the primary tool for the evaluation of thoracic lymph nodes with a potential for metastatic involvement. Given the diverse anatomical positions of numerous lymph nodes within the thorax, EBUS-TBNA (endobronchial ultrasound transbronchial needle aspiration) must be performed repetitively for each lymph node, as there are numerous lymph nodes localized in various anatomical positions within the thorax. Cytological results obtained from EBUS-TBNA (endobronchial ultrasound transbronchial needle aspiration) sampling influence therapeutic decisions, especially in the selection of patients for curative treatment [4,5].

Limitations in the sensitivity and specificity of preoperative lymph node detection, using computed tomography (CT) and positron emission tomography/computed tomography (PET/CT), emphasize the importance and need for the future improvement of the accurate identification of thoracic lymph node metastases with EBUS-TBNA [6,7]. During lung cancer staging, each target lymph node must be repeatedly localized and sampled with the highest precision to ensure the correct final lymph node stage [4,5]. EBUS-TBNA results are operator-dependent, leading to varying rates of cytological success across studies [8,9,10,11,12]. The suboptimal quality of current lymph node evaluation with EBUS-TBNA is partially indicated by the frequency of postoperative nodal upstaging (10–20%) and downstaging (10%) among surgically treated lung cancer patients [13,14,15]. To prevent futile surgeries and to be able to select the most effective cancer treatment for each patient, methods to improve lymph node evaluation and sampling with EBUS-TBNA are needed.

During EBUS, grayscale, Doppler, and elastography images can normally be displayed [16]. However, real-time assessments of macro- and microanatomy and tissue characteristics using these modalities can be challenging in the case of suboptimal imaging. Poor contact between the probe and the inner surface of the airways and artifacts generated by interposed cartilage and lung tissue tend to compromise the image quality. During EBUS, Doppler signals may be used to assess the vascularity of a lymph node or blood vessel [17]. Elastography can help in the identification of lymph nodes by providing qualitative or semi-quantitative measures of tissue elasticity [18]. One challenge is the varying subjective operator-dependent evaluation of Doppler and elastography, which can result in potential discrepancies in interpreting lymph nodes and blood vessels, as well as difficulties in localizing lymph node stations [17,18].

Artificial intelligence (AI) is a promising solution that could overcome challenges related to EBUS imaging and image segmentation in particular and has the potential for real-life, intraoperative use. The aim of this study was to detect, distinguish, and segment mediastinal lymph nodes and blood vessels using a novel U-Net architecture-based approach in EBUS images and evaluate the feasibility, degree of precision performance, and clinical functionality of the method.

## 2. Related Work

Due to recent advancements in lung cancer treatment, the use of EBUS has expanded. EBUS has now become a fundamental method in the staging and molecular profiling of lung cancer, with a significant impact on clinical decisions [2,3]. Consequently, new potential applications for AI’s integration into EBUS have emerged [19,20].

Using innovative image guidance methods, such as virtual bronchoscopy navigation (VBN) and electromagnetic tracking, in EBUS can help localize lymph nodes through optimal route planning and simultaneous position control. However, none of these methods are able to distinguish between different structures within an ultrasound image [21,22,23,24,25]. Certain image guidance systems involve segmentation, which is the delineation and anatomical localization of specific regions or structures of interest within medical images. In EBUS, the main structures of interest are typically the target lymph nodes for TBNA sampling and the blood vessels that serve as anatomical landmarks [26,27]. The segmentation of EBUS images has been used to enhance virtual bronchoscopy navigation and electromagnetic navigation [28]. Zang et al. presented an image-guided EBUS bronchoscopy system that generates a virtual EBUS view from a CT scan, which is then registered to live EBUS probe views [29,30,31]. Their method requires the identification of a region of interest (ROI) in the EBUS images with automated segmentation based on traditional image-processing techniques [28]. In some cases, the creation of an ROI requires multiple user interventions to select seed points within the EBUS image, suggesting that the method is not yet ready for clinical use [28].

Deep neural networks (DNNs), particularly the U-Net architecture proposed by Roenneberger et al., have shown significantly improved segmentation performance for various medical imaging modalities, including ultrasound [32,33,34,35]. Given the increasing use and complexity of EBUS procedures in clinical practice, the application of DNNs in EBUS images should be a highly interesting area of research [19,20,36,37,38,39]. However, only a subset of previous studies have involved automatic methods for the segmentation of ultrasound images. A study by Li et al. that included various segmentation models (such as U-Net, attention U-Net, R2U-Net, and attention R2U-Net) compared ROI segmentation with alternative techniques, such as grayscale probe, Doppler, and elastography images [19]. Other studies have proposed lymph node segmentation to aid in the classification of benign versus malignant lymph nodes based on sonographic features in EBUS images [36,39]. These AI-driven approaches aim to improve the success rate and tissue adequacy of EBUS-guided biopsies. AI-augmented EBUS has been used to support the diagnosis of malignant tissue, with variable results. However, further development of AI-assisted EBUS is needed to improve its clinical outcomes, visual interpretation, and diagnostic accuracy [20]. Improved image processing, such as new segmentation techniques, is crucial and is, therefore, the primary focus of our research.

## 3. Materials and Methods

### 3.1. Study Population and EBUS Procedure

Patients referred for EBUS-TBNA due to enlarged mediastinal and hilar lymph nodes were prospectively enrolled without randomization. This study received approval from the Regional Committees for Medical and Health Sciences Research Ethics (REK) Norway (identifier 240245 (approval date 14 April 2021) and 588006 (approval date 4 April 2023)) and the Local Data Access Committees (identifier 2021/3210-19442/2021 (approval date 21 June 2021) and 2023/1540-20710/2023 (approval date 4 July 2023)). Additionally, it was registered at ClinicalTrials.gov (identifier NCT05739331 (approval date/first posted 22 February 2023)).

### 3.2. Preoperative

All patients underwent standard preoperative clinical evaluations with clinical examinations, pulmonary function tests, and contrast-enhanced computed tomography (CT) of the chest and abdomen.

### 3.3. Intraoperative

**Figure 1:**
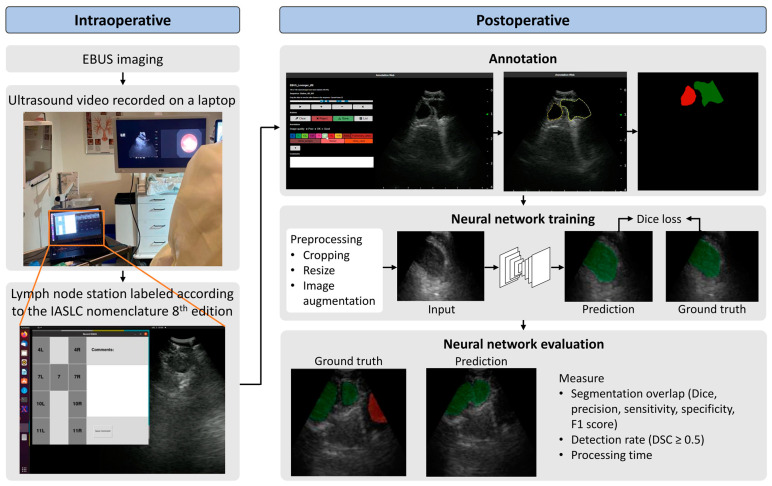
The study workflow, including both intraoperative and postoperative steps. Intraoperatively, the EBUS videos were recorded on a laptop. Lymph node stations were labeled in real-time on the laptop screen. Postoperatively, static images were selected for annotating, identifying, and marking lymph nodes and blood vessels. The annotated data were then used to train a deep neural network (U-Net) model. The performance of the model was evaluated on unseen data. The green color represents labeled lymph nodes, while the red color represents blood vessels.

EBUS-TBNA was conducted in accordance with regional standards and involved conscious sedation with midazolam and alfentanil. Following an initial inspection with a flexible bronchoscope, a BF UC19OF ultrasound bronchoscope (Olympus, Tokyo, Japan) was used for EBUS. EBUS imaging was performed using a 10 MHz frequency and a depth of 40 mm. Ultrasound videos obtained from the EBUS processor (EU-ME2, Olympus, Tokyo, Japan) were recorded on a laptop computer using a video grabber (AV.io, Epiphan Video, Palo Alto, CA, USA). EBUS was systematically used to visualize and record images at lymph node stations 4L, 4R, 7L, 7R, 10L, 10R, 11L, and 11R according to the IASLC 8th edition with Mountain–Dresler nomenclature [26,27]. Lymph node station 7 is a single station but was differentiated into 7R and 7L to distinguish between imaging on the left and right sides of the main carina. The recordings were labeled with the lymph node station intraoperatively on a laptop computer running an in-house developed software for this purpose (Figure 1). Two pulmonologists with more than 500 EBUS procedures of experience conducted all the study acquisitions [40].

### 3.4. Postoperative

The open-source software Annotation Web was employed for annotating structures in the EBUS videos [41]. The EBUS expert selected static images from the EBUS videos and annotated lymph nodes and vessels assisted by a spline segmentation technique (Figure 1) [42]. The annotation process was conducted by two experienced pulmonologists who were skilled in EBUS and EBUS image interpretation. They used a predefined annotation manual designed for this study, including positioned control points along the borders of the lymph nodes and blood vessels. They only included identifiable structures within the EBUS images. Separate splines were used in image frames with multiple structures, and overlap between the splines was avoided. All lymph nodes and blood vessels in each selected image were named according to the IASLC 8th edition with Mountain–Dresler nomenclature [26,27]. The images were exported from Annotation Web in PNG format.

### 3.5. Neural Network Architecture, Model Training, and Evaluation

#### 3.5.1. Training Scheme and Architecture

To train the segmentation model, we initially cropped all extraneous information from the images, leaving a uniform rectangular area encompassing the entire ultrasound sector, which was then resized to 256 × 256 pixels. Any segmentations outside the ultrasound sector were removed from the segmentation masks. During training, several image augmentations were applied. The dataset was randomly divided into training, validation, and test sets. A neural network based on the U-Net architecture was trained to segment the images [43]. The U-Net architecture selected was adapted by Leclerc et al. for the fast and accurate segmentation of the heart in echocardiography and uses a lower number of convolutions to achieve real-time performance [33]. The model was trained for 200 complete passes via the dataset, known as epochs, where each epoch consisted of processing batches of eight EBUS images. To optimize the model´s learning, we employed the Adam optimizer set at a learning rate of 0.001. Dice loss was chosen as the loss function. Early stopping with a patience of 20 epochs was used.

#### 3.5.2. Model Evaluation

To evaluate the model´s segmentation performance, we compared the predicted segmentations pixel-wise to the ground truth from the expert annotations for each class. All the reported metrics were from the evaluation in the test fold of the dataset. True and false positives and negatives were, thus, defined per pixel in each image. We used the following per-class evaluation metrics: the Dice similarity coefficient (DSC), precision, sensitivity, specificity, the F1 score, and detection (DSC > 50%). Further elaboration on these metrics can be found in detail in Table 1.

The processing time per image was reported in milliseconds (ms) and encompassed input/output operations, segmentation, and image display. To estimate the processing time, one warm-up run was conducted, followed by ten test runs of the segmentation pipeline on an EBUS video sequence containing ~250 images. These tests were performed on a laptop computer equipped with a CPU (Intel^®^ Core™ i7-10850H, Intel Corporation, Santa Clara, CA, USA) and a GPU (NVIDIA Quadro RTX 4000, NVIDIA Corporation, Santa Clara, CA, USA), using the image-processing framework FAST (version 5.6.0) with OpenVINO (version 2021.4.2) for inference [44].

## 4. Results

In total, the experts annotated 1161 EBUS images from 40 patients, including 1307 annotated lymph nodes and 800 annotated blood vessels across all anatomical lymph node stations. The distribution of the ultrasound images between lymph node stations 4L, 4R, 7L, 7R, 10L, 10R, 11L, and 11R is displayed in Table 2. Out of these, 882 images from 30 patients and 145 images from 5 patients were used for training and validation. A set of 134 images from five patients were kept separate from training and used for testing.

**Figure 2:**
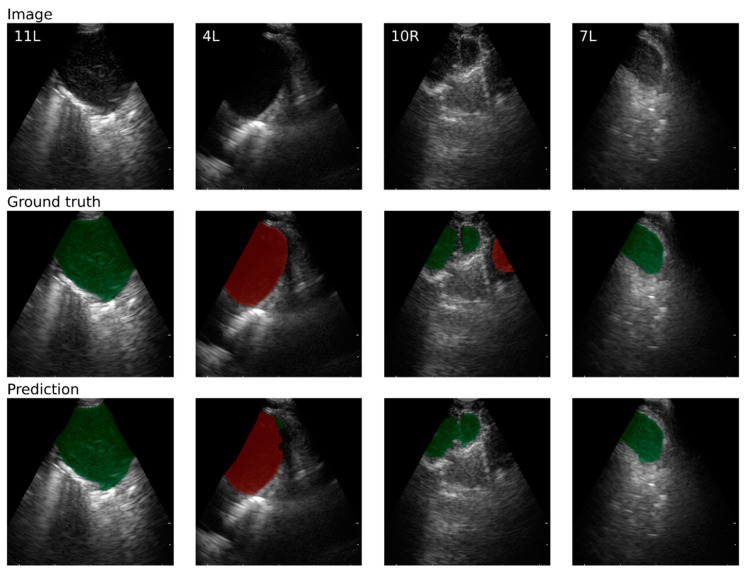
EBUS images (**top**) as well as ground-truth (**center**) and predicted (**bottom**) labels for lymph node stations/levels 11L, 4L, 10R, and 7L, respectively. Green represents labeled lymph nodes, and red represents blood vessels.

Figure 2 displays example EBUS images from four different lymph node stations, together with the expert annotations (ground truth) and segmentations predicted by the network.

**Figure 3:**
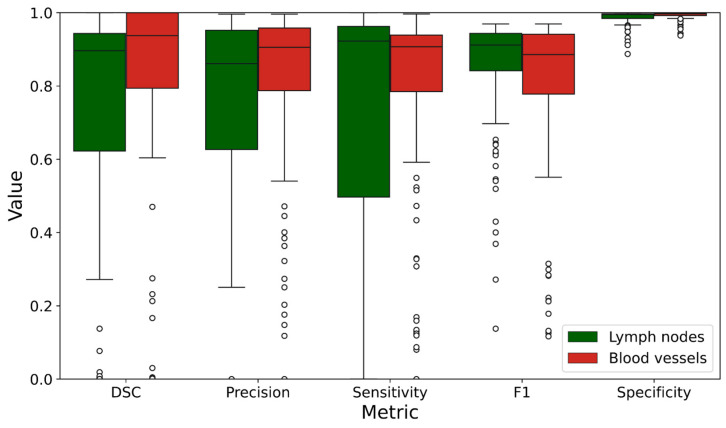
Segmentation metrics/parameters for lymph nodes (LNs, green) and blood vessels (BVs, red) in the test dataset. The boxes display the median, 25th percentile, and 75th percentile. The whiskers show the 5th and 95th percentiles. Outliers are marked with black circles.

Table 3 presents the respective mean ± standard deviation (SD) values for the automatic segmentation of lymph nodes and blood vessels. The segmentation performance for lymph nodes and blood vessels is presented in Figure 3.

In the images in the test set that contained a single instance of a lymph node or blood vessel, we established a cut-off DSC of 0.5 to consider the instance as detected. Using this approach, the model successfully detected 87 out of 89 lymph nodes (98%) and 44 out of 69 blood vessels (64%).

The average processing and segmentation run-time per ultrasound image was 55 ± 1 ms (mean ± SD) on a laptop equipped with a CPU and a GPU.

## 5. Discussion

The present human study demonstrated the feasibility of augmented EBUS with DNN-driven segmentation of the most clinically important anatomical structures within EBUS images. The primary focus was the automated detection and segmentation of mediastinal lymph nodes and blood vessels. The presented AI tool was able to distinguish and segment lymph nodes and vessels within the same model, allowing for real-time analysis, which is crucial for clinical use. The comparative results for the segmentation overlap (measured by the DSC) of lymph nodes and blood vessels were 0.71 and 0.76, respectively. These results highlight the potential of DNNs to assist bronchoscopists in the interpretation of EBUS images. The average processing and segmentation run-time per ultrasound image was 55 ± 1 ms (mean ± SD). While most EBUS processors operate at rates of 20–30 images per second or higher, our system processes approximately 18 images per second. This slight delay is unlikely to be perceptible to a bronchoscopist during EBUS in real time [45].

The comparable image-guided EBUS bronchoscopy system developed by Zang et al. integrated CT-based virtual EBUS views with live EBUS views [29,30,31]. The ROIs selected from the EBUS images were segmented using a technique previously described by the same group [28]. In a prospective study, they found that less than half of the targeted lymph nodes could be segmented fully automatically, while the segmentation of the remaining lymph nodes required user intervention (semi-automatic segmentation) [30]. The average time spent on lymph node segmentation during EBUS was 18.1 ± 14.6 s. Our DNN-based method for EBUS segmentation was substantially faster (55 ± 1 ms), providing fully automatic augmentation without requiring any user intervention. Moreover, the presented approach resulted in a high lymph node detection rate exceeding 95%.

Another potential application of DNNs in EBUS images is the automatic classification into benign or malignant lymph nodes, which is of great interest in future cancer-staging procedures [19,20,36,37,38,39]. In some previous studies, lymph nodes were manually mapped [20]. As an example, Li et al. automatically segmented lymph nodes using the U-Net architecture, achieving the highest DSC of 0.854 ± 0.0251 for ROI segmentation in all three ultrasound modes, grayscale images, Doppler, and elastography [19]. In their study, the majority of the segmented lymph nodes were located in lymph node stations 4R (33.67%) and 7 (34.01%). In contrast, our study included the systematic mapping of seven lymph node stations (Table 2). Systematic mapping is essential because clinical guidelines require the mapping of stations beyond 4R and 7, including the challenging mapping of stations like 10L and 4L, which we acknowledge as more intricate [4,5]. Furthermore, unlike the segmentation methods by Zang et al. and Li et al., our approach automatically distinguishes between lymph nodes and blood vessels, resulting in a DSC of 0.71 for lymph nodes and 0.76 for blood vessels.

Other published studies using DNNs for EBUS segmentation [36,39] do not provide quantitative measures for the segmentation performance or seem to assess the overlap between manual annotations performed in EBUS images and automatic segmentations. One main advantage of our method is the well-established segmentation technique used. Also, this study provides quantitative measures for segmentation performance that can be used by others for comparison.

In the presented study, the observed specificity was high (Table 3/Figure 3). This was probably affected by an abundance of background pixels within the ultrasound images used for the model’s development, leading the model to predict that the given pixels belonged to the background in 98.7% of cases. The majority of pixels represented true negatives, indicating a low rate of false-positive landmark detection by the model. The combination of a high detection rate (95%) and high specificity demonstrates that the presented network could be well suited for the accurate detection of genuine lymph nodes.

The standard deviation was high for several metrics (Table 3/Figure 3). Variation was expected due to the diversity among the recorded images. In some cases, the DSC approached one, marking the successful identification of the complete lymph node. However, in other cases, only certain parts of the lymph node or only one out of two lymph nodes were accurately identified.

Regarding the differentiation of lymph node station 7 into 7R and 7L, Table 2 shows four images labeled only as “station 7” in the training dataset. This was probably caused by human error during image labeling. Due to the low number of affected images, this was unlikely to have had any impact on the segmentation performance.

Our study had several strengths. First, it seamlessly integrated a software-only solution running on a laptop into existing systems for EBUS imaging. Second, it appeared without disrupting the workflow, and EBUS could be performed using standard bronchoscopy equipment and a conventional set-up in the bronchoscopy suite. By identifying blood vessels alongside lymph nodes, our method can enhance positional awareness, support the correct identification of mediastinal structures, and potentially enhance the user´s ability to localize the targeted lymph node. All study procedures and recordings were consistently performed by two bronchoscopists using the same methodology. The systematic imaging (mapping) and labeling of lymph nodes were performed according to the state-of-the-art nomenclature and clinical guidelines (Figure 1) [4,5,26]. There were few images and annotations from station 10L (Table 2). This was mainly due to the limited number and suboptimal image quality of 10L nodes among the included patients, which is in line with the observations of others [46]. The image annotations were conducted based on predefined criteria. To ensure the precise identification of lymph node stations and structures, the lymph node stations were labeled intraoperatively, whereas manual segmentation was performed after the procedure. Even though this study included patients with enlarged mediastinal and hilar lymph nodes, regardless of the final diagnosis, the heterogeneous nature of the lymph node characteristics reflects the real challenges faced by bronchoscopists in everyday practice. Furthermore, for DNN-based modeling, it is important that the training data cover the expected normal variation when the method is subjected to testing.

There were some study limitations. The network´s predictions differed from the ground truth in several cases, as represented by the outliers in Figure 3. Manual annotation was more challenging in the deeper parts of the ultrasound images, where structures were more poorly defined due to decreased resolution and more artifacts. Consequently, the annotations tended to emphasize structures near the EBUS probe while omitting deeper structures (Figure 2). An example can be seen in Figure 2 depicting lymph node station 10R. As a result, the prediction was wrongly assessed as a false positive in such cases. To minimize potential sources of error and bias, predefined criteria were established for the annotation process.

For the training of the DNN model, the most suitable EBUS images had to be selected from the recording, and annotations from two experienced bronchoscopists were used. Due to the exploratory nature of this study, a sample size estimate was not available. The training and validation of the DNN were conducted with a relatively small number of patients. Still, during the study period, we observed that the improvement in model performance from adding more patients to the study population became smaller. This suggests that, at some point, other pre-, intra-, and postoperative adjustments (e.g., adjusting the image depth to a smaller size), or the use of different model architectures (e.g., adding recurrent neural network (RNN) layers, such as long short-term memory (LSTM)), may be required to improve model performance. Since bronchoscopists may interpret EBUS images differently, even with predefined annotation criteria, it would be beneficial to validate the segmentation performance against inter-observer variability in the future. Furthermore, external validation using data from other clinics or EBUS manufacturers could enhance quality control.

The current study demonstrates that the real-time automatic segmentation of EBUS images can assist in the localization and detection of important mediastinal structures. The presented method could improve landmark recognition during EBUS-guided sampling from thoracic lymph nodes, with the potential to improve the sampling precision and reduce complication rates. This software-only solution is easily accessible and requires no extra time, resources, or user intervention. Thus, the experimental AI platform and software should have a clear potential for clinical use in minimally invasive lung cancer diagnosis and staging, as well as in endoscopy training.

As part of this study, we introduced a new dataset with EBUS images that have never been used for AI research before. The presented method and data could serve as a basis for further refinements and could also have transfer value to other ultrasound-based procedures. A topic of particular future interest will be the use of DNNs to identify the positions of structures in ultrasound images relative to other imaging modalities, such as CT or PET.

## 6. Conclusions

The present human study showed that EBUS-AI using a novel U-Net architecture-based approach was able to automatically detect and segment mediastinal lymph nodes and blood vessels. The method´s performance was good, and it was feasible and fast, enabling real-time automatic labeling. Our future objectives include enhancing the segmentation quality and the further development of software for the intraoperative labeling of lymph nodes and vessels, as well as classifying lymph node stations during EBUS-TBNA for improved sampling guidance.

## Figures and Tables

**Table 1:** Details of the metrics used to evaluate the performance of the segmentation model.

Metric	Formula	Description
Dice similarity coefficient (DSC)	$\frac{2|GT\cap P|}{\left|GT\right|+|P|}$	Measures’ overlap between the ground truth (GT) and predicted (P) segmentations.
Precision	$\frac{TP}{TP+FP}$	The ratio of the number of pixels correctly predicted to belong to the class (TP: true-positive prediction) to the total number of pixels predicted to belong to the class (TP + FP: false-positive prediction).
Sensitivity (recall)	$\frac{TP}{TP+FN}$	The ratio of the number of pixels correctly predicted to belong to the class (TP) to the true number of pixels belonging to the class (TP + FN: false-negative prediction).
Specificity	$\frac{TN}{TN+FP}$	The ratio of the number of pixels correctly predicted not to belong to the class (TN: true-negative predictions) to the number of pixels that do not belong to the class (TN + FP).
F1	$2\times \frac{\left(Precision\times Sensitivity\right)}{\left(Precision+Sensitivity\right)}$	The harmonic mean of precision and sensitivity.
Detection	DSC>0.5	For images with a single lymph node or blood vessel, the lymph node or blood vessel was counted as detected if DSC > 0.5.

**Table 2:** Distribution of images from each lymph node station.

	4L	4R	7L	7R	7	10L	10R	11L	11R	Sum
Variable					n (%)					n (%)
Training	149 (16.9)	150 (17.0)	129 (14.6)	142 (16.1)	4 (0.5)	18 (2.0)	109 (12.4)	78 (8.8)	103 (11.7)	882 (100)
Validation	31 (21.4)	30 (20.7)	18 (12.4)	13 (9.0)	(0.0)	(0.0)	14 (9.7)	18 (12.4)	21 (14.5)	145 (100)
Testing	29 (21.6)	21 (15.7)	26 (19.4)	19 (14.2)	(0.0)	(0.0)	8 (6.0)	14 (10.4)	17 (12.7)	134 (100)

Data are presented as the number (n) and fraction (%) of images from each lymph node station in the training, validation, and test datasets, respectively.

**Table 3:** Network performance in the segmentation of lymph nodes and blood vessels.

	Lymph Nodes		Blood Vessels	
	Mean	SD	Mean	SD
DSC	0.713	0.347	0.758	0.376
Precision	0.694	0.362	0.824	0.221
Sensitivity	0.711	0.380	0.797	0.251
F1	0.847	0.160	0.806	0.214
Specificity	0.987	0.018	0.992	0.011

Data are presented as means and standard deviation (SD). DSC (Dice similarity coefficient).

## Data Availability

The datasets presented in this article are not readily available because the data are part of an ongoing study. Requests to access the datasets should be directed to Øyvind Ervik.
